# Supplementary material for: Genome-wide association studies of global Mycobacterium tuberculosis resistance to 13 antimicrobials in 10,228 genomes identify new resistance mechanisms
Source: PLoS Biol. 2022 Aug 9;20(8):e3001755. doi: 10.1371/journal.pbio.3001755 (PMC9363015; doi:10.1371/journal.pbio.3001755)
Supplement: S1 Acknowledgements — (DOCX) [file pbio.3001755.s003.docx]

**Members of the CRyPTIC consortium (in alphabetical order)**

Sarah G Earle^4^, Daniel J Wilson^4^, Ivan Barilar^29^, Simone Battaglia^1^, Emanuele Borroni^1^, Angela Pires Brandao^2,3^, Alice Brankin^4^, Andrea Maurizio Cabibbe^1^, Joshua Carter^5^, Darren Chetty^30^, Daniela Maria Cirillo^1^, Pauline Claxton^6^, David A Clifton^4^, Ted Cohen^7^, Jorge Coronel^8^, Derrick W Crook^4^, Viola Dreyer^29^, Vincent Escuyer^9^, Lucilaine Ferrazoli^3^, Philip W Fowler^4^, George Fu Gao^10^, Jennifer Gardy^11^, Saheer Gharbia^12^, Kelen Teixeira Ghisi^3^, Arash Ghodousi^1,13^, Ana Luíza Gibertoni Cruz^4^, Louis Grandjean^33^, Clara Grazian^14^, Ramona Groenheit^44^, Jennifer L Guthrie^15,16^, Wencong He^10^, Harald Hoffmann^17,18^, Sarah J Hoosdally^4^, Martin Hunt^19,4^, Zamin Iqbal^19^, Nazir Ahmed Ismail^20^, Lisa Jarrett^21^, Lavania Joseph^20^, Ruwen Jou^22^, Priti Kambli^23^, Rukhsar Khot^23^, Jeff Knaggs^19,4^, Anastasia Koch^24^, Donna Kohlerschmidt^9^, Samaneh Kouchaki^4,25^, Alexander S Lachapelle^4^, Ajit Lalvani^26^, Simon Grandjean Lapierre^27^, Ian F Laurenson^6^, Brice Letcher^19^, Wan-Hsuan Lin^22^, Chunfa Liu^10^, Dongxin Liu^10^, Kerri M Malone^19^, Ayan Mandal^28^, Mikael Mansjö^44^, Daniela Matias^21^, Graeme Meintjes^24^, Flávia de Freitas Mendes^3^, Matthias Merker^29^, Marina Mihalic^18^, James Millard^30^, Paolo Miotto^1^, Nerges Mistry^28^, David Moore^31,8^, Kimberlee A Musser^9^, Dumisani Ngcamu^20^, Hoang Ngoc Nhung^32^, Stefan Niemann^29, 48^, Kayzad Soli Nilgiriwala^28^, Camus Nimmo^33^, Max O’Donnell^49^, Nana Okozi^20^, Rosangela Siqueira Oliveira^3^, Shaheed Vally Omar^20^, Nicholas Paton^34^, Timothy EA Peto^4^, Juliana Maira Watanabe Pinhata^3^, Sara Plesnik^18^, Zully M Puyen^35^, Marie Sylvianne Rabodoarivelo^36^, Niaina Rakotosamimanana^36^, Paola MV Rancoita^13^, Priti Rathod^21^, Esther Robinson^21^, Gillian Rodger^4^, Camilla Rodrigues^23^, Timothy C Rodwell^37,38^, Aysha Roohi^4^, David Santos-Lazaro^35^, Sanchi Shah^28^, Thomas Andreas Kohl^29^, Grace Smith^21,12^, Walter Solano^8^, Andrea Spitaleri^1,13^, Philip Supply^39^, Adrie JC Steyn^30^, Utkarsha Surve^23^, Sabira Tahseen^40^, Nguyen Thuy Thuong Thuong^32^, Guy Thwaites^32,4^, Katharina Todt^18^, Alberto Trovato^1^, Christian Utpatel^29^, Annelies Van Rie^41^, Srinivasan Vijay^42^, Timothy M Walker^4,32^, A Sarah Walker^4^, Robin Warren^43^, Jim Werngren^44^, Maria Wijkander^44^, Robert J Wilkinson^45,46,26^, Penelope Wintringer^19^, Yu-Xin Xiao^22^, Yang Yang^4^, Zhao Yanlin^10^, Shen-Yuan Yao^20^, Baoli Zhu^47^

**Institutions**

1 IRCCS San Raffaele Scientific Institute, Milan, Italy

2 Oswaldo Cruz Foundation, Rio de Janeiro, Brazil

3 Institute Adolfo Lutz, São Paulo, Brazil

4 University of Oxford, Oxford, UK

5 Stanford University School of Medicine, Stanford, USA

6 Scottish Mycobacteria Reference Laboratory, Edinburgh, UK

7 Yale School of Public Health, Yale, USA

8 Universidad Peruana Cayetano Heredia, Lima, Perú

9 Wadsworth Center, New York State Department of Health, Albany, USA

10 Chinese Center for Disease Control and Prevention, Beijing, China

11 Bill & Melinda Gates Foundation, Seattle, USA

12 UK Health Security Agency, London, UK

13 Vita-Salute San Raffaele University, Milan, Italy

14 University of New South Wales, Sydney, Australia

15 The University of British Columbia, Vancouver, Canada

16 Public Health Ontario, Toronto, Canada

17 SYNLAB Gauting, Munich, Germany

18 Institute of Microbiology and Laboratory Medicine, IMLred, WHO-SRL Gauting, Germany

19 EMBL-EBI, Hinxton, UK

20 National Institute for Communicable Diseases, Johannesburg, South Africa

21 UK Health Security Agency, Birmingham, UK

22 Taiwan Centers for Disease Control, Taipei, Taiwan

23 Hinduja Hospital, Mumbai, India

24 University of Cape Town, Cape Town, South Africa

25 University of Surrey, Guildford, UK

26 Imperial College, London, UK

27 Université de Montréal, Canada

28 The Foundation for Medical Research, Mumbai, India

29 Research Center Borstel, Borstel, Germany

30 Africa Health Research Institute, Durban, South Africa

31 London School of Hygiene and Tropical Medicine, London, UK

32 Oxford University Clinical Research Unit, Ho Chi Minh City, Viet Nam

33 University College London, London, UK

34 National University of Singapore, Singapore

35 Instituto Nacional de Salud, Lima, Perú

36 Institut Pasteur de Madagascar, Antananarivo, Madagascar

37 FIND, Geneva, Switzerland

38 University of California, San Diego, USA

39 Univ. Lille, CNRS, Inserm, CHU Lille, Institut Pasteur de Lille, U1019 - UMR 9017 - CIIL - Center for Infection and Immunity of Lille, F-59000 Lille, France

40 National TB Reference Laboratory, National TB Control Program, Islamabad, Pakistan

41 University of Antwerp, Antwerp, Belgium

42 University of Edinburgh, Edinburgh, UK

43 Stellenbosch University, Cape Town, South Africa

44 Public Health Agency of Sweden, Solna, Sweden

45 Wellcome Centre for Infectious Diseases Research in Africa, Cape Town, South Africa

46 Francis Crick Institute, London, UK

47 Institute of Microbiology, Chinese Academy of Sciences, Beijing, China

48 German Center for Infection Research (DZIF), Hamburg-Lübeck-Borstel-Riems, Germany

49 Colombia University Irving Medical Center, New York, USA
