# Supplementary material for: Genome-wide association studies of global Mycobacterium tuberculosis resistance to 13 antimicrobials in 10,228 genomes identify new resistance mechanisms
Source: PLoS Biol. 2022 Aug 9;20(8):e3001755. doi: 10.1371/journal.pbio.3001755 (PMC9363015; doi:10.1371/journal.pbio.3001755)
Supplement: S1 Fig — The red dashed line indicates the ECOFF, measurements to the left of the ECOFF are considered sensitive, and those to the right are considered resistant. AMI, amikacin; BDQ, bedaquiline; CFZ, clofazimine; DLM, delamanid; ECOFF, epidemiological cutoff; EMB, ethambutol; ETH, ethionamide; GWAS, genome-wide association studies; INH, isoniazid; KAN, kanamycin; LEV, levofloxacin; LZD, linezolid; MIC, minimum inhibitory concentration; MXF, moxifloxacin; RFB, rifabutin; RIF, rifampicin. (PDF) [file pbio.3001755.s004.pdf]

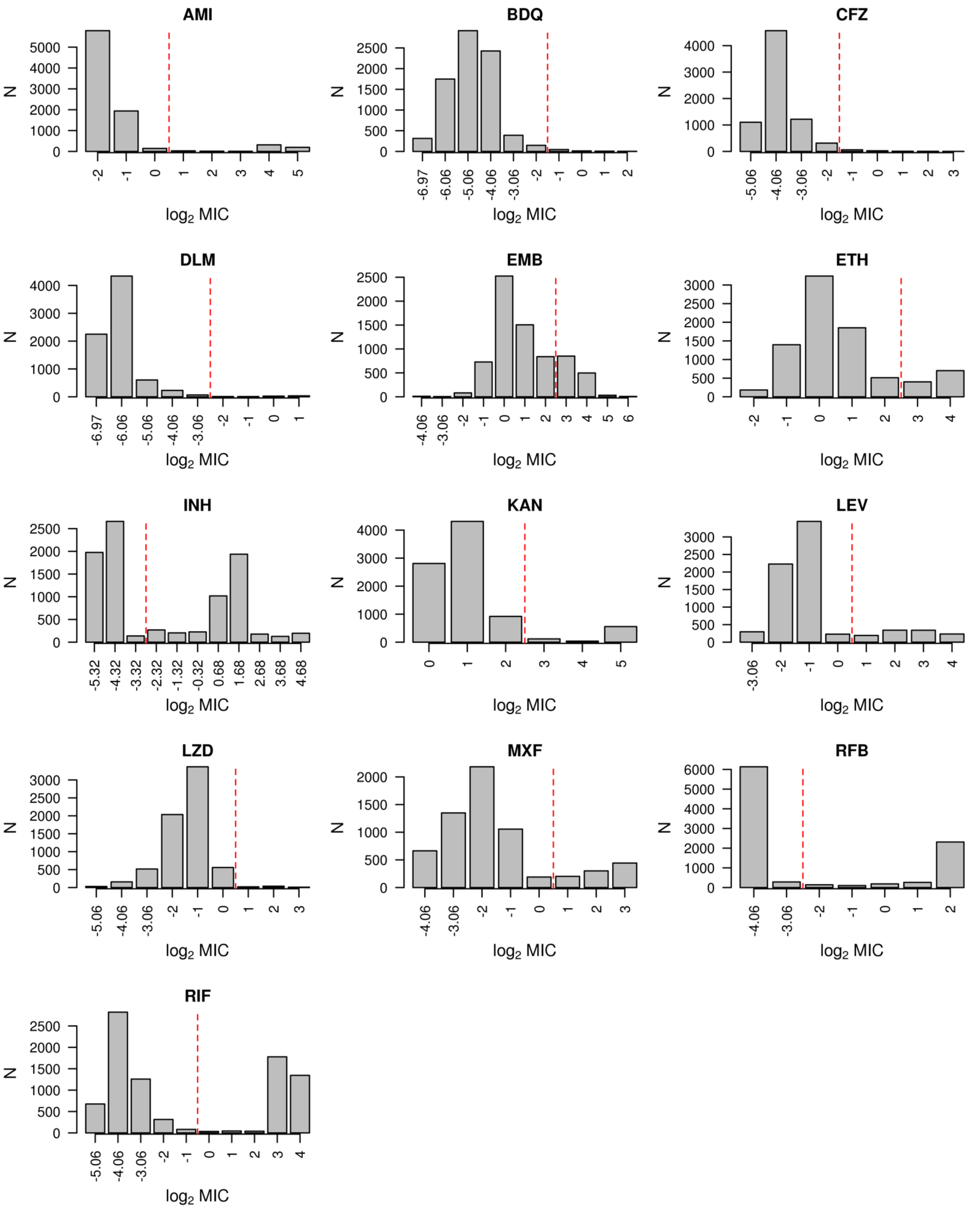

S1 Fig. Distributions of the $\log_2$ MIC measurements for all 13 drugs in the GWAS analyses, amikacin (AMI), bedaquiline (BDQ), clofazimine (CFZ), delamanid (DLM), ethambutol (EMB), ethionamide (ETH), isoniazid (INH), kanamycin (KAN), levofloxacin (LEV), linezolid (LZD), moxifloxacin (MXF), rifabutin (RFB) and rifampicin (RIF). The red dashed line indicates the ECOFF, measurements to the left of the ECOFF are considered sensitive, those to the right are considered resistant.
