# Supplementary material for: Genome-wide association studies of global Mycobacterium tuberculosis resistance to 13 antimicrobials in 10,228 genomes identify new resistance mechanisms
Source: PLoS Biol. 2022 Aug 9;20(8):e3001755. doi: 10.1371/journal.pbio.3001755 (PMC9363015; doi:10.1371/journal.pbio.3001755)
Supplement: S2 Fig — Sample heritability estimates and 95% CIs are shown for the 13 drugs, DLM, CFZ, LZD), BDQ, MXF, LEV, KAN, EMB, ETH, AMI, RIF, INH, and RFB. When estimating heritability of the same phenotype, the oligopeptide and oligonucleotide estimates are very similar. AMI, amikacin; BDQ, bedaquiline; CI, confidence interval; CFZ, clofazimine; DLM, delamanid; EMB, ethambutol; ETH, ethionamide; INH, isoniazid; KAN, kanamycin; LEV, levofloxacin; LZD, linezolid; MIC, minimum inhibitory concentration; MXF, moxifloxacin; RFB, rifabutin; RIF, rifampicin. (PDF) [file pbio.3001755.s005.pdf]

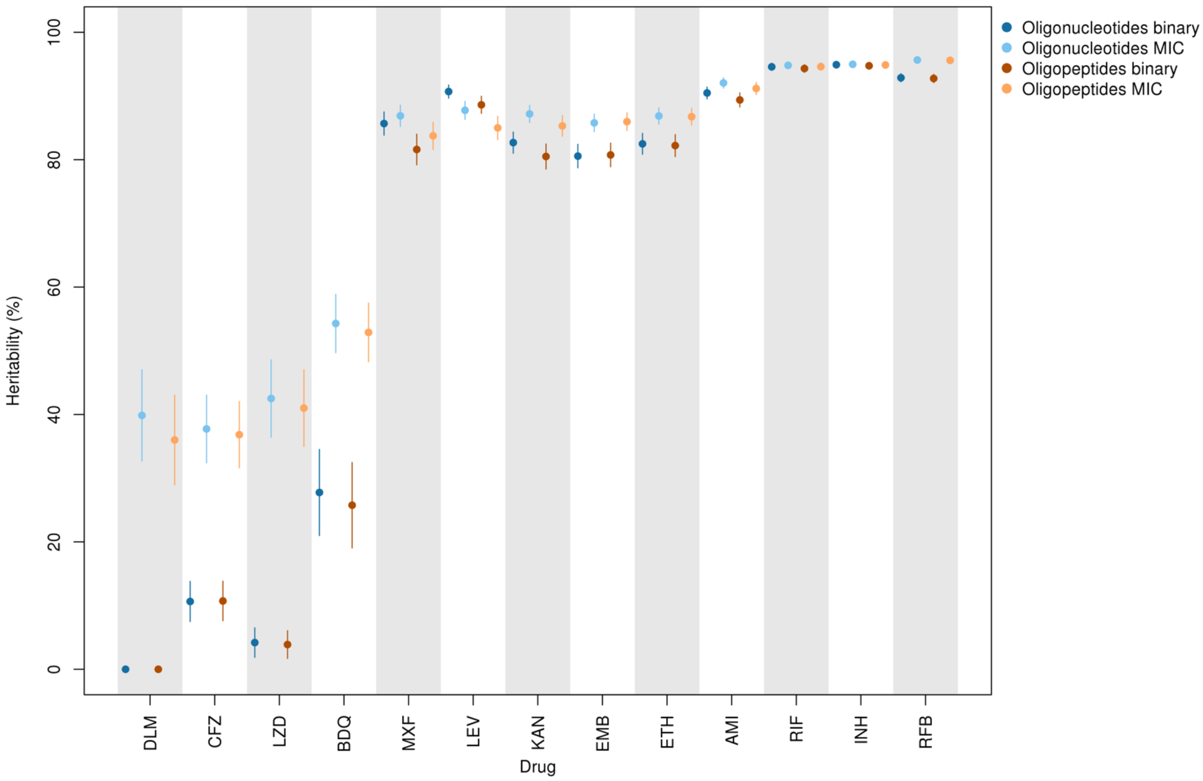

S2 Fig. Oligopeptide and oligonucleotide sample heritability estimates for binary resistant vs. sensitive phenotypes compared to semi-quantitiative MIC phenotypes. Sample heritability estimates and 95% confidence intervals are shown for the 13 drugs, DLM (delamanid), clofazimine (CFZ), linezolid (LZD), bedaquiline (BDQ), moxifloxacin (MXF), levofloxacin (LEV), kanamycin (KAN), ethambutol (EMB), ethionamide (ETH), amikacin (AM), rifampicin (RIF), isoniazid (INH), rifabutin (RFB). When estimating heritability of the same phenotype, the oligopeptide and oligonucleotide estimates are very similar.
