# Supplementary material for: Genome-wide association studies of global Mycobacterium tuberculosis resistance to 13 antimicrobials in 10,228 genomes identify new resistance mechanisms
Source: PLoS Biol. 2022 Aug 9;20(8):e3001755. doi: 10.1371/journal.pbio.3001755 (PMC9363015; doi:10.1371/journal.pbio.3001755)
Supplement: S3 Fig — Comparing the empirical distribution of p-values to the expected distribution under the null hypothesis for the drugs AMI, BDQ, CFZ, DLM, EMB, ETH, INH, and KAN. Oligopeptides in the orange (MAF < 0.1%) were not initially analysed, only used for signal interpretation. AMI, amikacin; BDQ, bedaquiline; CFZ, clofazimine; DLM, delamanid; EMB, ethambutol; ETH, ethionamide; INH, isoniazid; KAN, kanamycin; MAF, minor allele frequency. (PDF) [file pbio.3001755.s006.pdf]

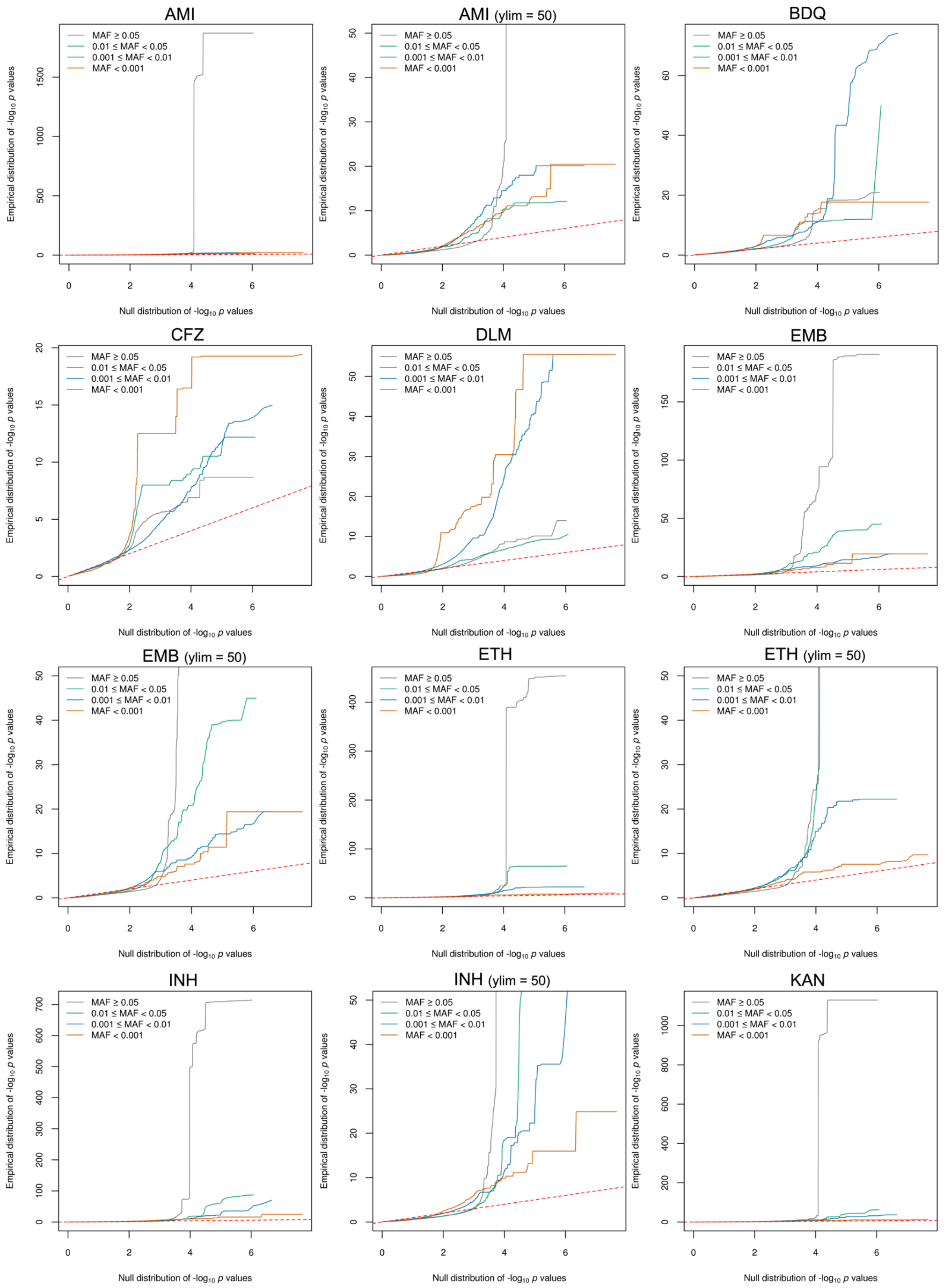

S3 Fig. QQ plots for the oligopeptide analyses, part A. Comparing the empirical distribution of p-values to the

expected distribution under the null hypothesis for the drugs amikacin (AMI), bedaquiline (BDQ), clofazimine (CFZ), delamanid (DLM), ethambutol (EMB), ethionamide (ETH), isoniazid (INH), kanamycin (KAN). Oligopeptides in the orange (MAF < 0.1%) were not initially analysed, only used for signal interpretation.
