# Supplementary material for: Genome-wide association studies of global Mycobacterium tuberculosis resistance to 13 antimicrobials in 10,228 genomes identify new resistance mechanisms
Source: PLoS Biol. 2022 Aug 9;20(8):e3001755. doi: 10.1371/journal.pbio.3001755 (PMC9363015; doi:10.1371/journal.pbio.3001755)
Supplement: S4 Fig — Comparing the empirical distribution of p-values to the expected distribution under the null hypothesis for the KAN, LEV, LZD, MXF, RFB, and RIF. Oligopeptides in the orange (MAF < 0.1%) were not initially analysed, only used for signal interpretation. KAN, kanamycin; LEV, levofloxacin; LZD, linezolid; MAF, minor allele frequency; MXF, moxifloxacin; RFB, rifabutin; RIF, rifampicin. (PDF) [file pbio.3001755.s007.pdf]

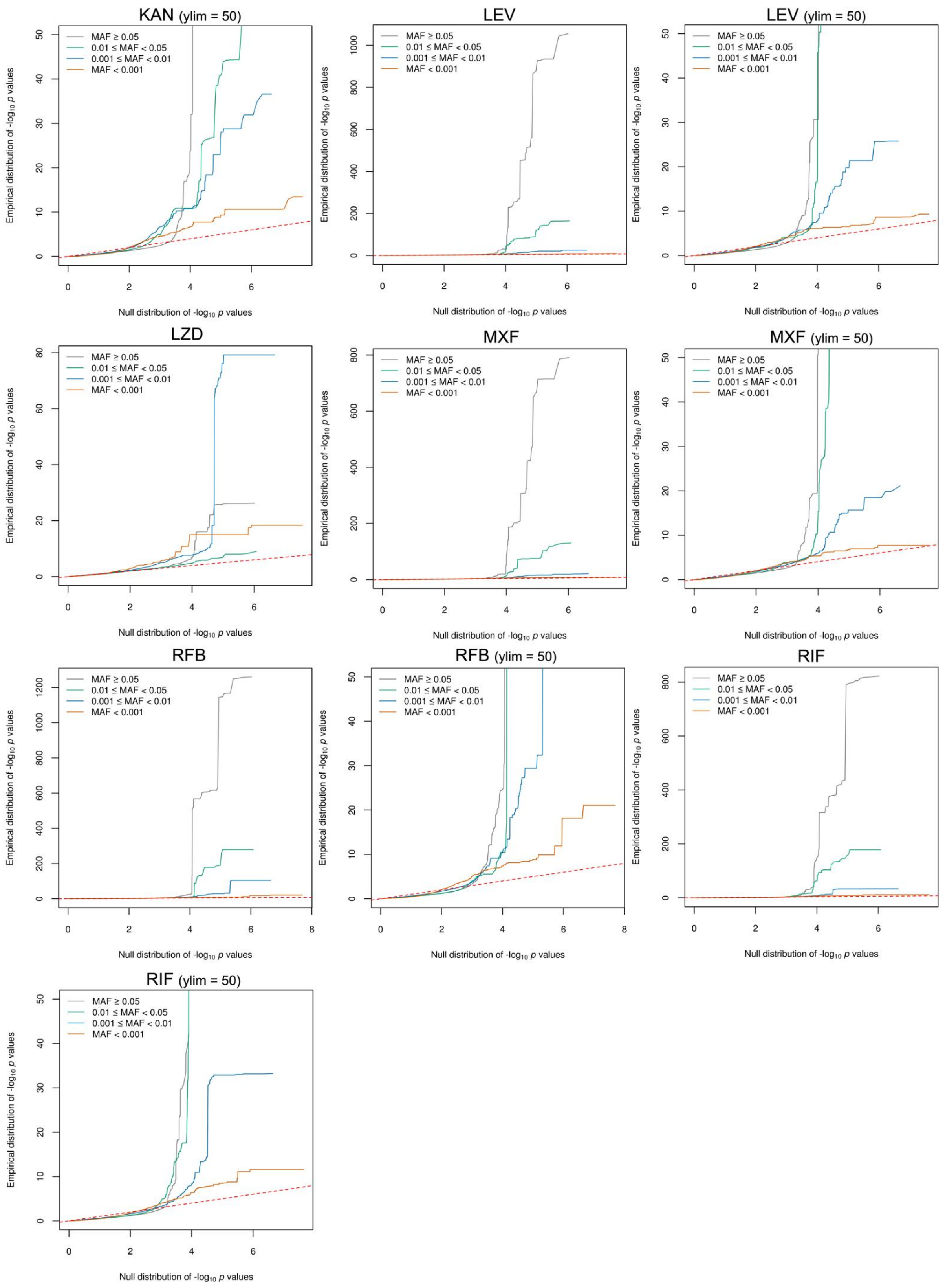

S4 Fig. QQ plots for the oligopeptide analyses, part B. Comparing the empirical distribution of p-values to the

expected distribution under the null hypothesis for the kanamycin (KAN), levofloxacin (LEV), linezolid (LZD), moxifloxacin (MXF), rifabutin (RFB) and rifampicin (RIF). Oligopeptides in the orange (MAF < 0.1%) were not initially analysed, only used for signal interpretation.
