# Supplementary material for: Genome-wide association studies of global Mycobacterium tuberculosis resistance to 13 antimicrobials in 10,228 genomes identify new resistance mechanisms
Source: PLoS Biol. 2022 Aug 9;20(8):e3001755. doi: 10.1371/journal.pbio.3001755 (PMC9363015; doi:10.1371/journal.pbio.3001755)
Supplement: S5 Fig — For many of the drugs, the most significant oligopeptides were associated with lower MIC. AMI, amikacin; BDQ, bedaquiline; CFZ, clofazimine; DLM, delamanid; EMB, ethambutol; ETH, ethionamide; INH, isoniazid; KAN, kanamycin; LEV, levofloxacin; LZD, linezolid; MIC, minimum inhibitory concentration; MXF, moxifloxacin; RFB, rifabutin; RIF, rifampicin. (PDF) [file pbio.3001755.s008.pdf]

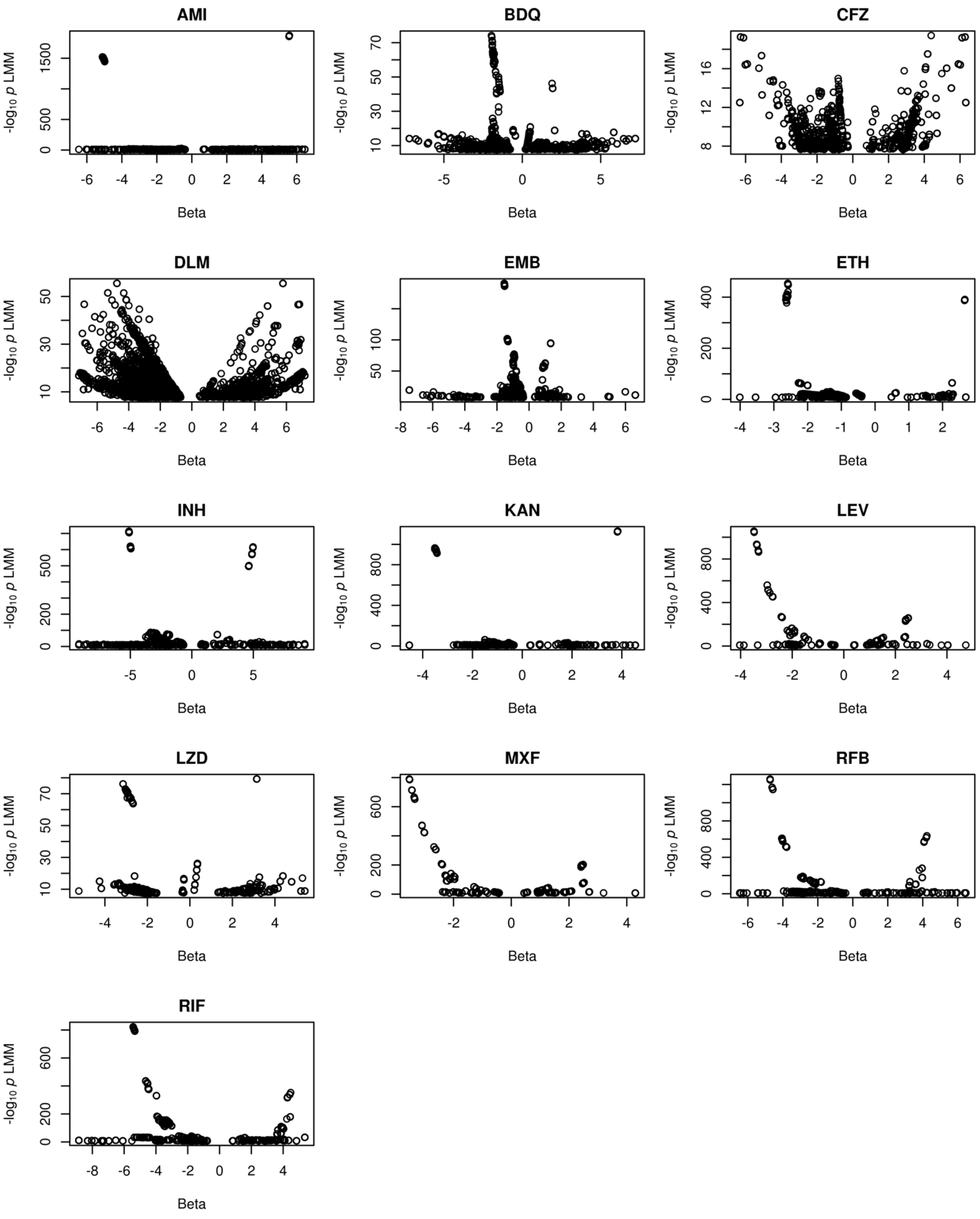

S5 Fig. Effect size (beta) estimates and $-\log_{10}$ *p*-values for all significant oligopeptide variants for each drug, amikacin (AMI), bedaquiline (BDQ), clofazimine (CFZ), delamanid (DLM), ethambutol (EMB), ethionamide (ETH), isoniazid (INH), kanamycin (KAN), levofloxacin (LEV), linezolid (LZD), moxifloxacin (MXF), rifabutin (RFB) and rifampicin (RIF). For many of the drugs, the most significant oligopeptides were associated with lower MIC.
