# Supplementary material for: Genome-wide association studies of global Mycobacterium tuberculosis resistance to 13 antimicrobials in 10,228 genomes identify new resistance mechanisms
Source: PLoS Biol. 2022 Aug 9;20(8):e3001755. doi: 10.1371/journal.pbio.3001755 (PMC9363015; doi:10.1371/journal.pbio.3001755)
Supplement: S6 Fig — On the left, the beta estimates are shown for all significant oligopeptides for the drugs the gene is causal for, on the right, the beta estimates are shown for the same gene, but for the drugs they are artefactually associated to. For many drugs, the beta estimate is lower when the gene is significant due to artefactual cross-resistance. Drug name abbreviations are as follows: AMI, BDQ, CFZ, DLM, EMB, ETH, INH, KAN, LEV, LZD, MXF, RFB, and RIF. AMI, amikacin; BDQ, bedaquiline; CFZ, clofazimine; DLM, delamanid; EMB, ethambutol; ETH, ethionamide; INH, isoniazid; KAN, kanamycin; LEV, levofloxacin; LZD, linezolid; MXF, moxifloxacin; RFB, rifabutin; RIF, rifampicin. (PDF) [file pbio.3001755.s009.pdf]

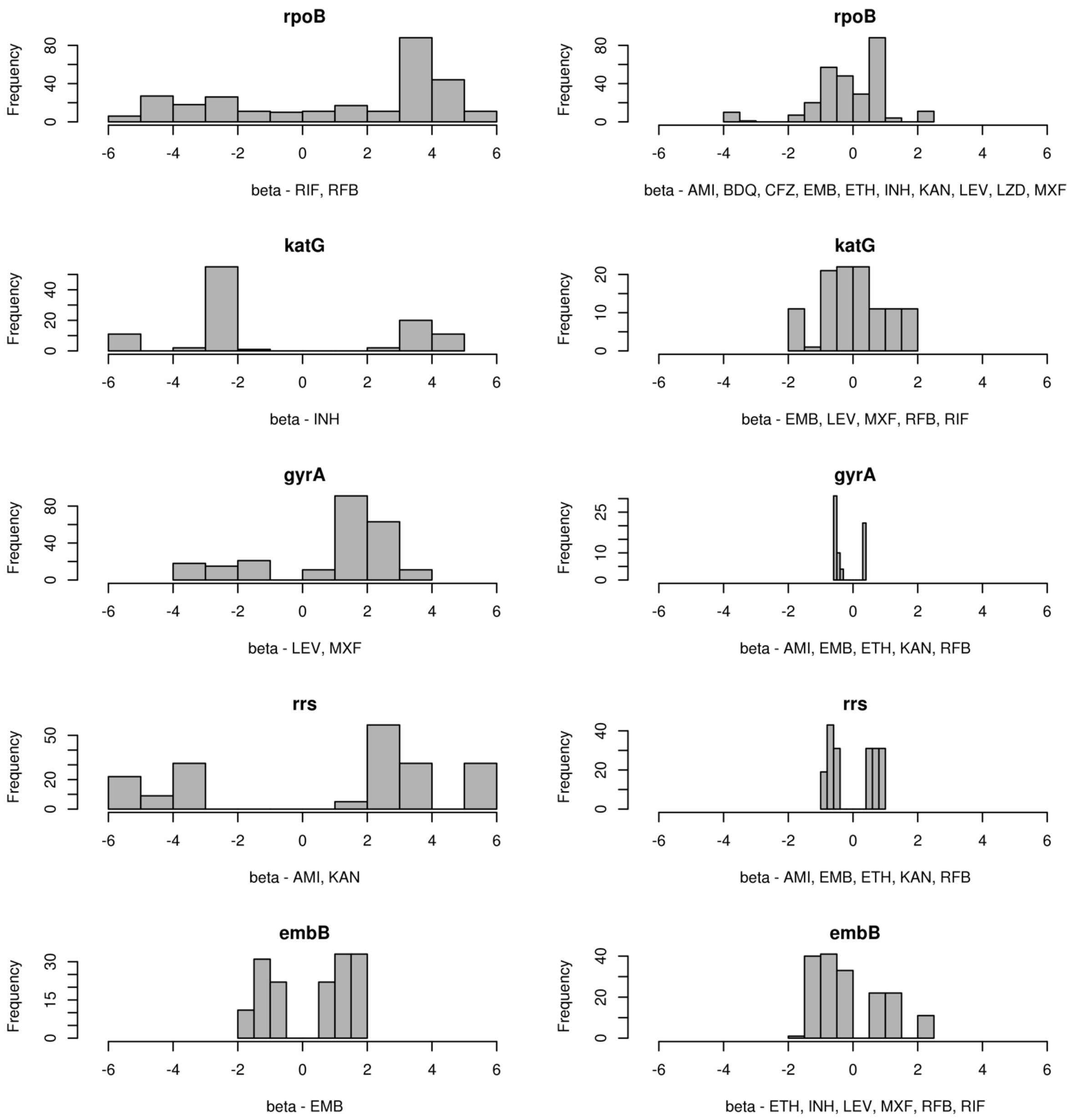

S6 Fig. Significant oligopeptide (*rpoB, katG, gyrA, embB*) and oligonucleotide (*rrs*) effect size (beta) estimates for known resistance genes plus the flanking 33 amino acids (oligopeptides) or 100 bases (oligonucelotides). On the left the beta estimates are shown for all significant oligopeptides for the drugs the gene is causal for, on the right the beta estimates are shown for the same gene, but for the drugs they are artefactually associated to. For many drugs, the beta estimate is lower when the gene is significant due to artefactual cross resistance. Drug name abbreviations are as follows: amikacin (AMI), bedaquiline (BDQ), clofazimine (CFZ), delamanid (DLM), ethambutol (EMB), ethionamide (ETH), isoniazid (INH), kanamycin (KAN), levofloxacin (LEV), linezolid (LZD), moxifloxacin (MXF), rifabutin (RFB) and rifampicin (RIF).
