# Supplementary material for: Genome-wide association studies of global Mycobacterium tuberculosis resistance to 13 antimicrobials in 10,228 genomes identify new resistance mechanisms
Source: PLoS Biol. 2022 Aug 9;20(8):e3001755. doi: 10.1371/journal.pbio.3001755 (PMC9363015; doi:10.1371/journal.pbio.3001755)
Supplement: S7 Fig — Manhattan plots showing the oligopeptide association results for the spoU coding region A ethambutol and B rifampicin, and oligonucleotide alignment plots showing close-ups of the significant region just downstream of spoU for C ethambutol and D rifampicin. The black dashed lines indicate the Bonferroni-corrected significance thresholds. In the Manhattan plots, oligopeptides are coloured by the reading frame that they align to, black for the correct reading frame for spoU. Oligopeptides assigned to the region but did not align using BLAST are shown in grey on the right-hand side of the plots. In the oligonucleotide alignment plots, the H37Rv reference codons are shown at the bottom of the figure, grey for an invariant site, coloured at variant site positions. The oligonucleotides that aligned to the region are plotted from least significant at the bottom to most significant at the top. The background colour of the oligonucleotides represents the direction of the b estimate, light grey when b < 0 (associated with lower MIC), dark grey when b > 0 (associated with higher MIC). Oligonucleotides are coloured by their amino acid residue at all variant positions. Oligonucleotides below the MAF threshold and not included in the analysis, but visualised here for signal interpretation, are marked by *s. The spoU stop codon is highlighted in red in the alignment plots. EMB, ethambutol; MAF, minor allele frequency; MIC, minimum inhibitory concentration; RIF, rifampicin. (PDF) [file pbio.3001755.s010.pdf]

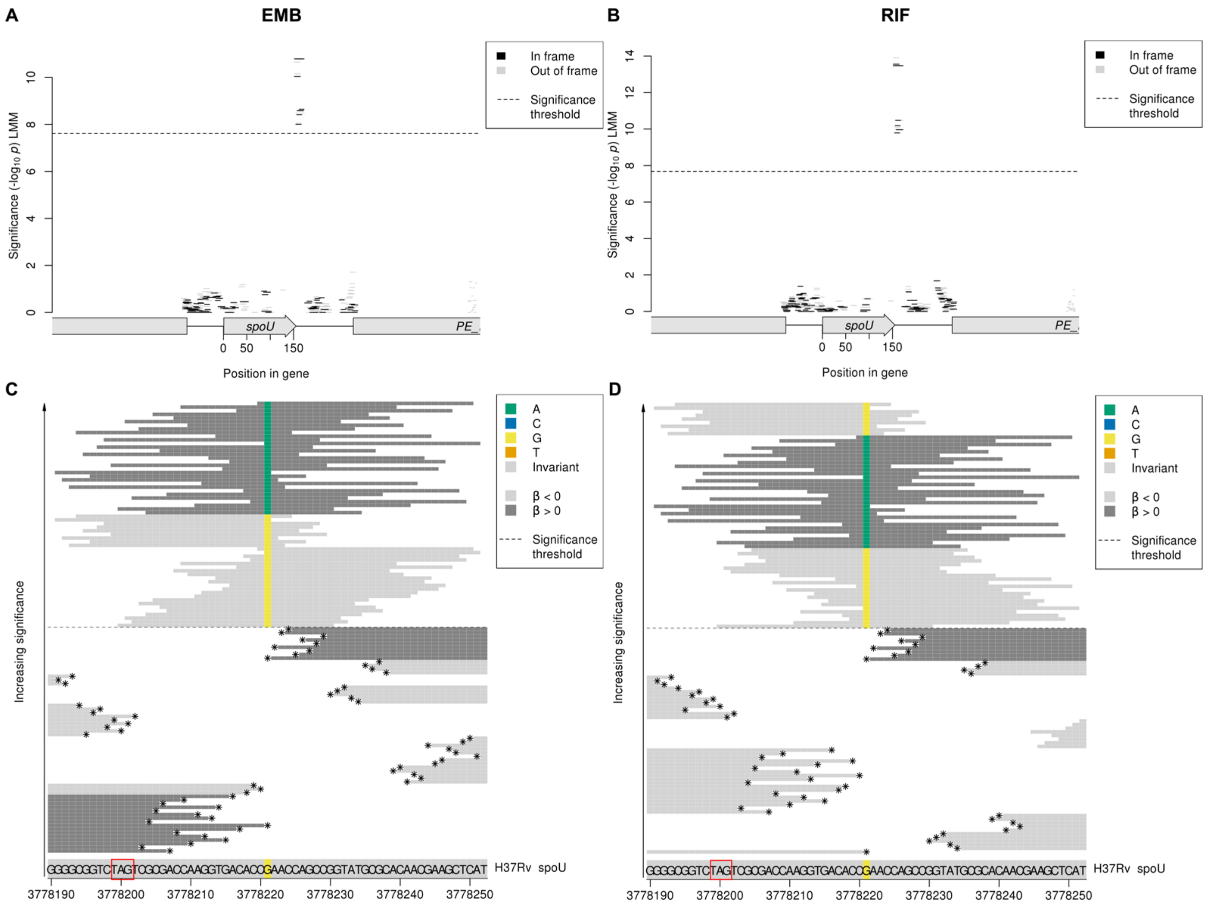

S7 Fig. Variants in *spoU* associated with ethambutol (EMB) and rifampicin (RIF) MIC. Manhattan plots showing the oligopeptide association results for the *spoU* coding region **A** ethambutol and **B** rifampicin, and oligonucleotide alignment plots showing close ups of the significant region just downstream of *spoU* for **C** ethambutol and **D** rifampicin. The black dashed lines indicate the Bonferroni-corrected significance thresholds. In the Manhattan plots, oligopeptides are coloured by the reading frame that they align to, black for the correct reading frame for *spoU*. Oligopeptides assigned to the region but did not align using BLAST are shown in grey on the right hand side of the plots. In the oligonucleotide alignment plots, the H37Rv reference codons are shown at the bottom of the figure, grey for an invariant site, coloured at variant site positions. The oligonucleotides that aligned to the region are plotted from least significant at the bottom to most significant at the top. The background colour of the oligonucleotides represents the direction of the b estimate, light grey when b < 0 (associated with lower MIC), dark grey when b > 0 (associated with higher MIC). Oligonucleotides are coloured by their amino acid residue at all variant positions. Oligonucleotides below the MAF threshold and not included in the analysis, but visualised here for signal interpretation, are marked by *s. The *spoU* stop codon is highlighted in red in the alignment plots.
