# Supplementary material for: Genome-wide association studies of global Mycobacterium tuberculosis resistance to 13 antimicrobials in 10,228 genomes identify new resistance mechanisms
Source: PLoS Biol. 2022 Aug 9;20(8):e3001755. doi: 10.1371/journal.pbio.3001755 (PMC9363015; doi:10.1371/journal.pbio.3001755)
Supplement: S8 Fig — Manhattan plots showing the association results for the Rv1219c coding region for the A oligopeptides and B oligonucleotides, and oligopeptide alignment plots showing close-ups of the significant region in Rv1219c for C oligopeptides present in 5 or more genomes in the full GWAS dataset and D oligopeptides present in at least 1 genome in the full GWAS dataset. The black dashed lines indicate the Bonferroni-corrected significance thresholds. In the Manhattan plots, oligopeptides are coloured by the reading frame that they align to, black for the correct reading frame for Rv1219c. Oligopeptides and nucleotides assigned to the region but did not align using BLAST are shown in grey on the right-hand side of the plots. In the oligopeptide alignment plots, the H37Rv reference codons are shown at the bottom of the figure, grey for an invariant site, coloured at variant site positions. The oligopeptides that aligned to the region are plotted from least significant at the bottom to most significant at the top. The background colour of the oligopeptides represents the direction of the b estimate, light grey when b < 0 (associated with lower MIC), dark grey when b > 0 (associated with higher MIC). Oligopeptides are coloured by their amino acid residue at all variant positions. Oligopeptides and nucleotides below the MAF threshold and not included in the analysis, but visualised here for signal interpretation, are marked by *s. GWAS, genome-wide association studies; MAF, minor allele frequency; MIC, minimum inhibitory concentration. (PDF) [file pbio.3001755.s011.pdf]

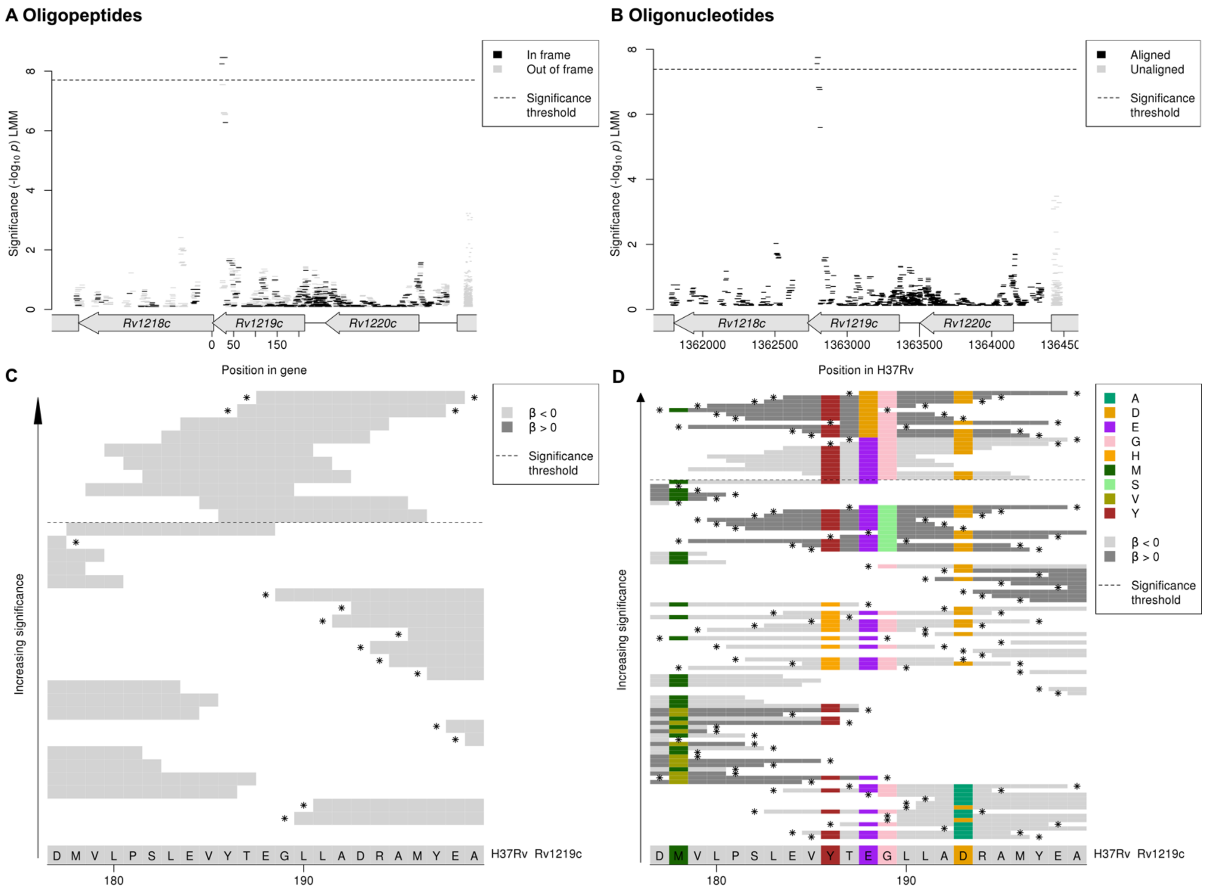


**S8 Fig.** Variants in *Rv1219c* associated with isoniazid MIC. Manhattan plots showing the association results for the *Rv1219c* coding region for the **A** oligopeptides and **B** oligonucleotides, and oligopeptide alignment plots showing close ups of the significant region in *Rv1219c* for **C** oligopeptides present in five or more genomes in the full GWAS dataset and **D** oligopeptides present in at least one genome in the full GWAS dataset. The black dashed lines indicate the Bonferroni-corrected significance thresholds. In the Manhattan plots, oligopeptides are coloured by the reading frame that they align to, black for the correct reading frame for *Rv1219c*. Oligo-peptides and nucleotides assigned to the region but did not align using BLAST are shown in grey on the right hand side of the plots. In the oligopeptide alignment plots, the H37Rv reference codons are shown at the bottom of the figure, grey for an invariant site, coloured at variant site positions. The oligopeptides that aligned to the region are plotted from least significant at the bottom to most significant at the top. The background colour of the oligopeptides represents the direction of the b estimate, light grey when b < 0 (associated with lower MIC), dark grey when b > 0 (associated with higher MIC). Oligopeptides are coloured by their amino acid residue at all variant positions. Oligo-peptides and nucleotides below the MAF threshold and not included in the analysis, but visualised here for signal interpretation, are marked by *s.
