# Supplementary material for: Genome-wide association studies of global Mycobacterium tuberculosis resistance to 13 antimicrobials in 10,228 genomes identify new resistance mechanisms
Source: PLoS Biol. 2022 Aug 9;20(8):e3001755. doi: 10.1371/journal.pbio.3001755 (PMC9363015; doi:10.1371/journal.pbio.3001755)
Supplement: S12 Fig — Manhattan plots showing the association results for the coding region for A cysA2 and B cysA3, and oligonucleotide alignment plots showing close-ups of the significant region for C cysA2 and D cysA3. The black dashed lines indicate the Bonferroni-corrected significance thresholds. The significant oligonucleotides that align to cysA2 and cysA3 are the same. In the Manhattan plots, oligopeptides are coloured by the reading frame that they align to, black for the correct reading frame for cysA2 or cysA3. Oligopeptides assigned to the region but did not align using BLAST are shown in grey on the right-hand side of the plot. In the oligonucleotide alignment plots, the H37Rv reference alleles are shown at the bottom of the figure, grey for an invariant site, coloured at variant site positions. The oligonucleotides that aligned to the region are plotted from least significant at the bottom to most significant at the top. The background colour of the oligonucleotides represents the direction of the b estimate, light grey when b < 0 (associated with lower MIC), dark grey when b > 0 (associated with higher MIC). Oligonucleotides are coloured by their allele at all variant positions. Oligonucleotides below the MAF threshold and not included in the analysis, but visualised here for signal interpretation, are marked by *s. The region that encodes the rhodanese characteristic signature in the N-terminal region is highlighted in red in the alignment plots. MAF, minor allele frequency; MIC, minimum inhibitory concentration. (PDF) [file pbio.3001755.s015.pdf]

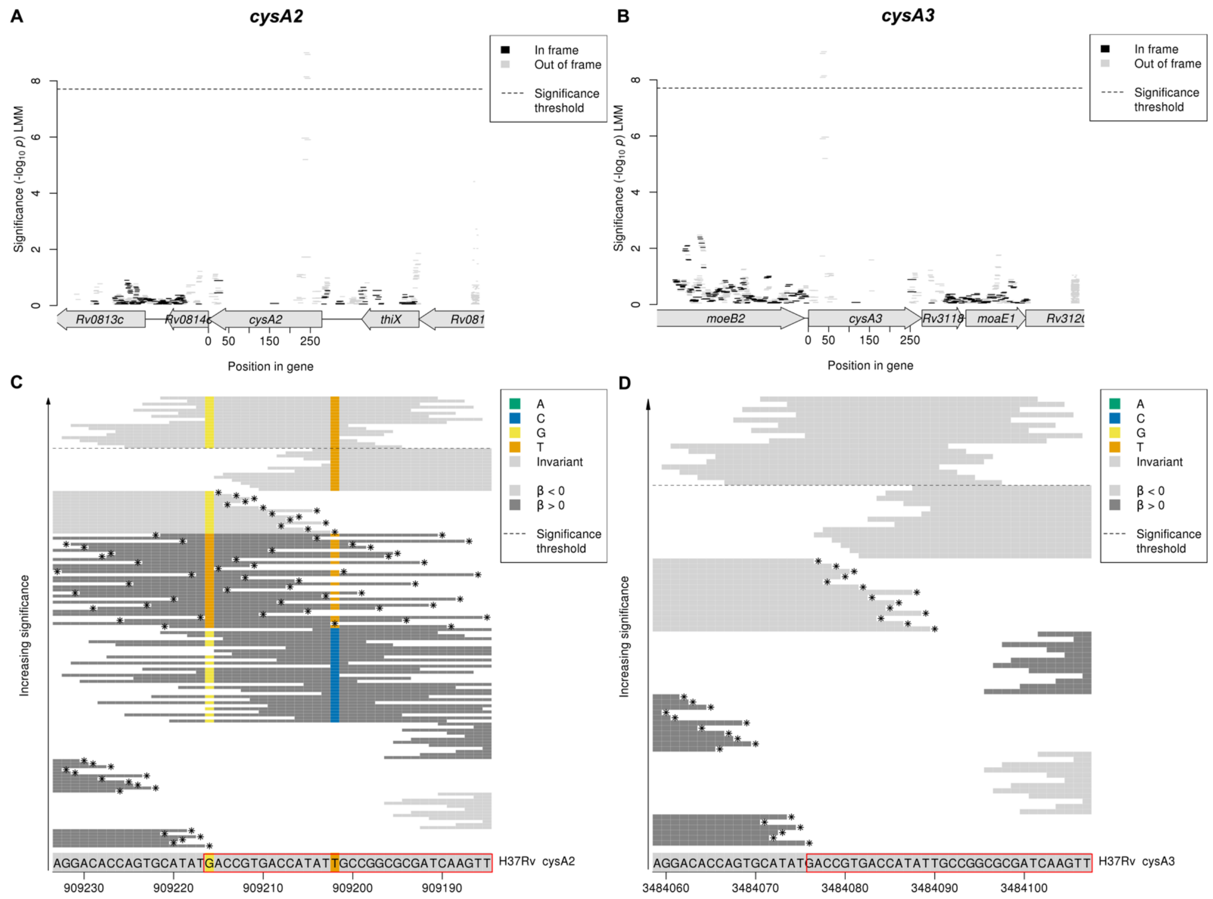

S12 Fig in *cysA2* and *cysA3* associated with rifabutin MIC. Manhattan plots showing the association results for the coding region for **A** *cysA2* and **B** cysA3, and oligonucleotide alignment plots showing close ups of the significant region for **C** *cysA2* **D** *cysA3*. The black dashed lines indicate the Bonferroni-corrected significance thresholds. The significant oligonucleotides that align to *cysA2* and *cysA3* are the same. In the Manhattan plots, oligopeptides are coloured by the reading frame that they align to, black for the correct reading frame for *cysA2* or *cysA3*. Oligopeptides assigned to the region but did not align using BLAST are shown in grey on the right hand side of the plot. In the oligonucleotide alignment plots, the H37Rv reference alleles are shown at the bottom of the figure, grey for an invariant site, coloured at variant site positions. The oligonucleotides that aligned to the region are plotted from least significant at the bottom to most significant at the top. The background colour of the oligonucleotides represents the direction of the b estimate, light grey when b < 0 (associated with lower MIC), dark grey whenb> 0 (associated with higher MIC). Oligonucleotides are coloured by their allele at all variant positions. Oligonucleotides below the MAF threshold and not included in the analysis, but visualised here for signal interpretation, are marked by *s. The region that encodes the rhodanese characteristic signature in the N-terminal region is highlighted in red in the alignment plots.
