# Supplementary material for: Genome-wide association studies of global Mycobacterium tuberculosis resistance to 13 antimicrobials in 10,228 genomes identify new resistance mechanisms
Source: PLoS Biol. 2022 Aug 9;20(8):e3001755. doi: 10.1371/journal.pbio.3001755 (PMC9363015; doi:10.1371/journal.pbio.3001755)
Supplement: S15 Fig — Manhattan plots showing the association results for the pknH coding region for the A oligopeptides and B oligonucleotides, and alignment plots showing close-ups of the significant region in pknH for the C oligopeptides and D oligonucleotides. The black dashed lines indicate the Bonferroni-corrected significance thresholds. In the Manhattan plots, oligopeptides are coloured by the reading frame that they align to, black for the correct reading frame for pknH. Oligopeptides and nucleotides assigned to the region but did not align using BLAST are shown in grey on the right-hand side of the plots. In the alignment plots, the H37Rv reference alleles are shown at the bottom of the figure, grey for an invariant site, coloured at variant site positions. The oligopeptides and nucleotides that aligned to the region are plotted from least significant at the bottom to most significant at the top. The background colour of the oligopeptides and nucleotides represents the direction of the b estimate, light grey when b < 0 (associated with lower MIC), dark grey when b > 0 (associated with higher MIC). Oligopeptides and nucleotides are coloured by their allele at all variant positions. Oligopeptides and nucleotides below the MAF threshold and not included in the analysis, but visualised here for signal interpretation, are marked by *s. MAF, minor allele frequency; MIC, minimum inhibitory concentration. (PDF) [file pbio.3001755.s018.pdf]

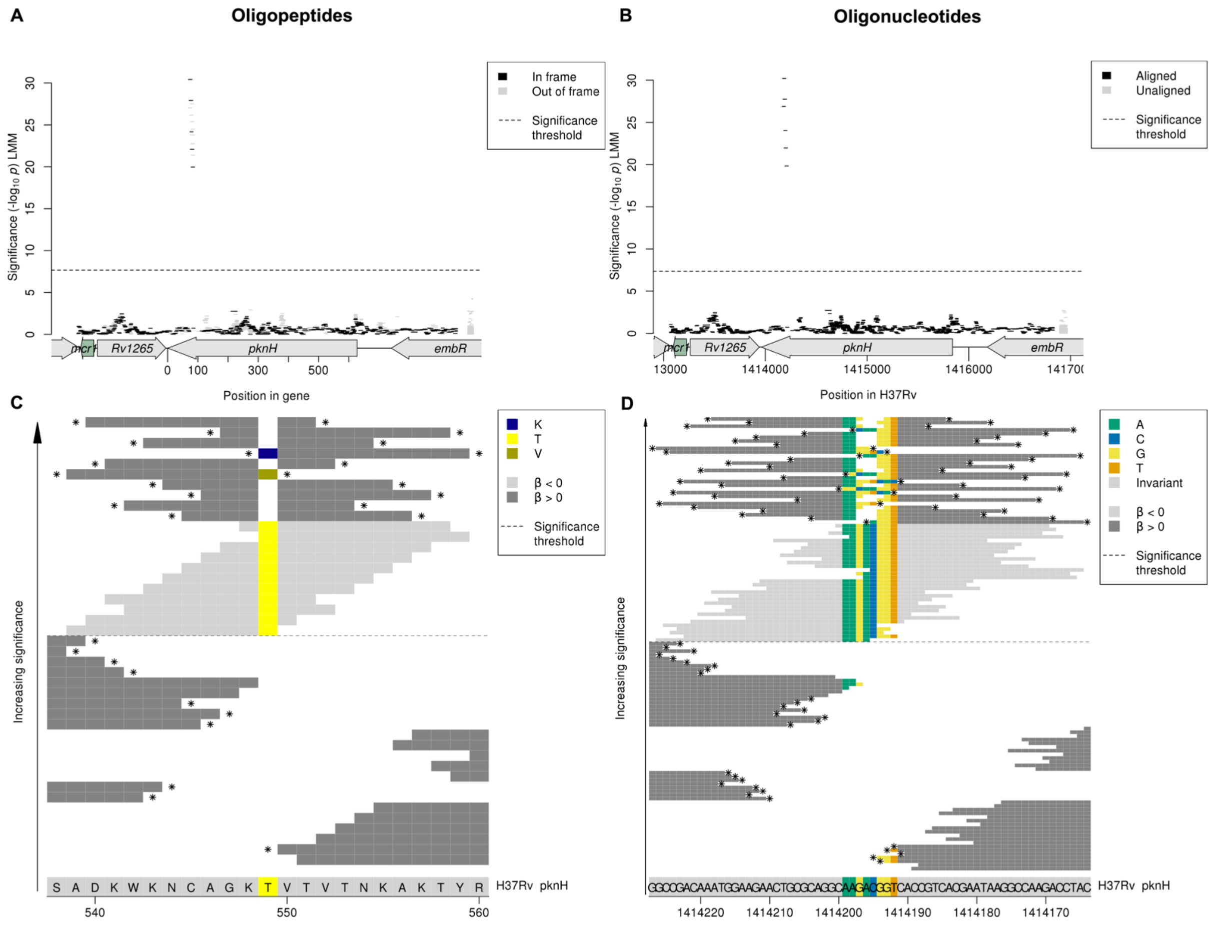

S15 Fig. Variants in *pknH* associated with delamanid MIC. Manhattan plots showing the association results for the *pknH* coding region for the **A** oligopeptides and **B** oligonucleotides, and alignment plots showing close ups of the significant region in *pknH* for the **C** oligopeptides **D** oligonucleotides. The black dashed lines indicate the Bonferroni-corrected significance thresholds. In the Manhattan plots, oligopeptides are coloured by the reading frame that they align to, black for the correct reading frame for *pknH*. Oligo-peptides and nucleotides assigned to the region but did not align using BLAST are shown in grey on the right hand side of the plots. In the alignment plots, the H37Rv reference alleles are shown at the bottom of the figure, grey for an invariant site, coloured at variant site positions. The oligo-peptides and nucleotides that aligned to the region are plotted from least significant at the bottom to most significant at the top. The background colour of the oligo-peptides and nucleotides represents the direction of the b estimate, light grey when b < 0 (associated with lower MIC), dark grey when b> 0 (associated with higher MIC). Oligo-peptides and nucleotides are coloured by their allele at all variant positions. Oligo-peptides and nucleotides below the MAF threshold and not included in the analysis, but visualised here for signal interpretation, are marked by *s.
