# Supplementary material for: Genome-wide association studies of global Mycobacterium tuberculosis resistance to 13 antimicrobials in 10,228 genomes identify new resistance mechanisms
Source: PLoS Biol. 2022 Aug 9;20(8):e3001755. doi: 10.1371/journal.pbio.3001755 (PMC9363015; doi:10.1371/journal.pbio.3001755)
Supplement: S16 Fig — Manhattan plots showing the association results for vapB20 for the A oligopeptides and B oligonucleotides, and C oligonucleotide alignment plot showing a close-up of the significant region just upstream of vapB20. The black dashed lines indicate the Bonferroni-corrected significance thresholds. In the Manhattan plots, oligopeptides are coloured by the reading frame that they align to, black for the correct reading frame for amiA2. Oligopeptides and nucleotides assigned to the region but did not align using BLAST are shown in grey on the right-hand side of the plots. In the oligonucleotide alignment plot, the H37Rv reference alleles are shown at the bottom of the figure, grey for an invariant site, coloured at variant site positions. The oligonucleotides that aligned to the region are plotted from least significant at the bottom to most significant at the top. The background colour of the oligonucleotides represents the direction of the b estimate, light grey when b < 0 (associated with lower MIC), dark grey when b > 0 (associated with higher MIC). Oligonucleotides are coloured by their allele at all variant positions. Oligopeptides and nucleotides below the MAF threshold and not included in the analysis, but visualised here for signal interpretation, are marked by *s. MAF, minor allele frequency; MIC, minimum inhibitory concentration. (PDF) [file pbio.3001755.s019.pdf]

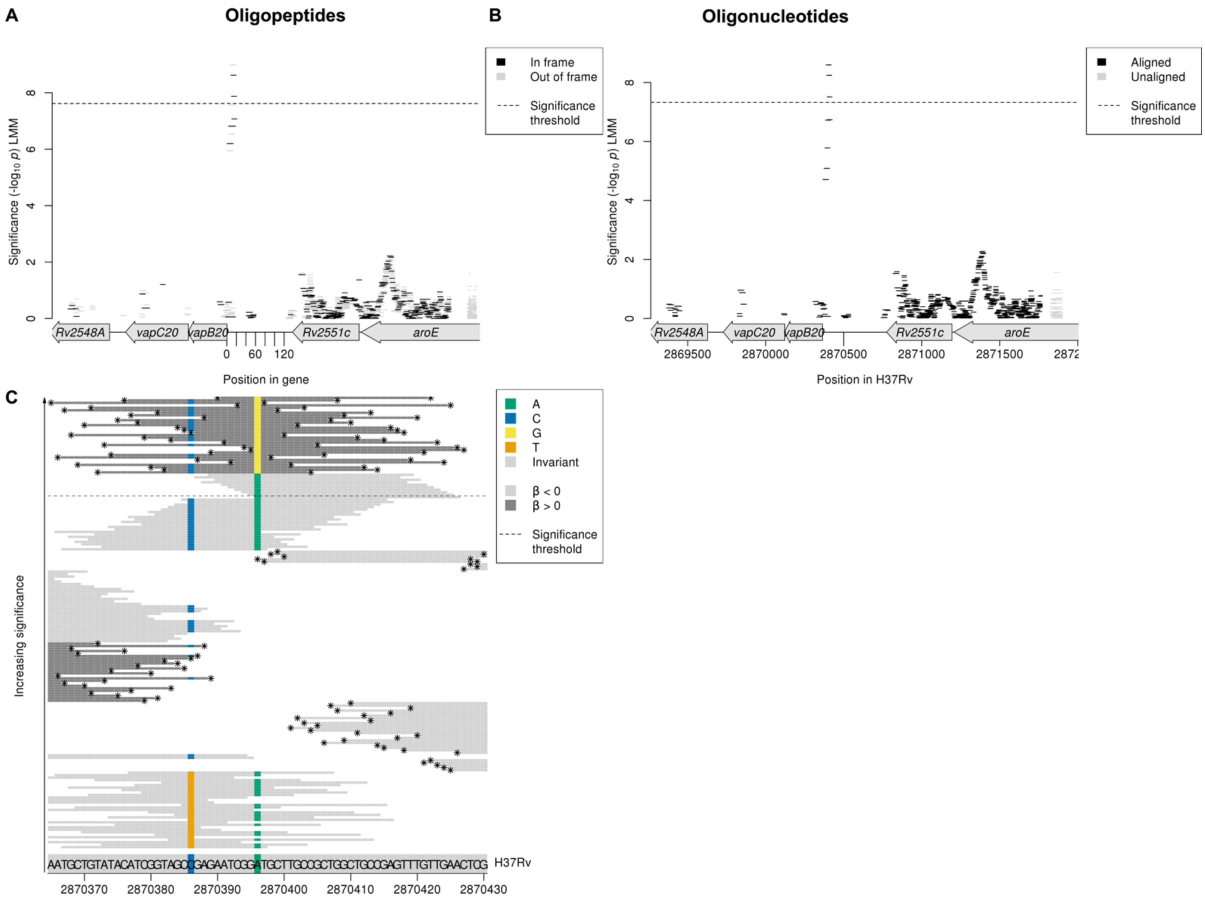

S16 Fig. Variants in *vapB20* associated with linezolid MIC. Manhattan plots showing the association results for *vapB20* for the **A** oligopeptides and **B** oligonucleotides, and **C** oligonucleotide alignment plot showing a close up of the significant region just upstream of *vapB20*. The black dashed lines indicates the Bonferroni-corrected significance thresholds. In the Manhattan plots, oligopeptides are coloured by the reading frame that they align to, black for the correct reading frame for *amiA2*. Oligo-peptides and nucleotides assigned to the region but did not align using BLAST are shown in grey on the right hand side of the plots. In the oligonucleotide alignment plot, the H37Rv reference alleles are shown at the bottom of the figure, grey for an invariant site, coloured at variant site positions. The oligonucleotides that aligned to the region are plotted from least significant at the bottom to most significant at the top. The background colour of the oligonucleotides represents the direction of the b estimate, light grey when b < 0 (associated with lower MIC), dark grey when b> 0 (associated with higher MIC). Oligonucleotides are coloured by their allele at all variant positions. Oligo-peptides and nucleotides below the MAF threshold and not included in the analysis, but visualised here for signal interpretation, are marked by *s.
