# Supplementary material for: Genome-wide association studies of global Mycobacterium tuberculosis resistance to 13 antimicrobials in 10,228 genomes identify new resistance mechanisms
Source: PLoS Biol. 2022 Aug 9;20(8):e3001755. doi: 10.1371/journal.pbio.3001755 (PMC9363015; doi:10.1371/journal.pbio.3001755)
Supplement: S1 Table — Sample heritability estimates and 95% CIs are shown for the 13 drugs. CI, confidence interval; MIC, minimum inhibitory concentration. (PDF) [file pbio.3001755.s020.pdf]

	Heritability estimates and 95% confidence intervals			
	**Oligopeptides MIC**	**Oligopeptides binary**	**Oligonucleotides MIC**	**Oligonucleotides binary**
**Delamanid**	36.0 (28.94-43.05)	0 (-0.49-0.49)	39.86 (32.69-47.02)	0 (-0.45-0.45)
**Clofazimine**	36.83 (31.62-42.04)	10.72 (7.61-13.83)	37.73 (32.41-43.05)	10.64 (7.49-13.8)
**Linezolid**	41 (34.98-47.01)	3.88 (1.7-6.06)	42.51 (36.44-48.58)	4.19 (1.87-6.51)
**Bedaquiline**	52.88 (48.26-57.5)	25.76 (19.04-32.47)	54.29 (49.71-58.86)	27.75 (20.99-34.52)
**Moxifloxacin**	83.74 (81.6-85.88)	81.6 (79.2-84.01)	86.88 (85.21-88.56)	85.67 (83.85-87.5)
**Levofloxacin**	85 (83.18-86.81)	88.61 (87.27-89.95)	87.75 (86.34-89.17)	90.69 (89.66-91.73)
**Kanamycin**	85.3 (83.68-86.92)	80.5 (78.53-82.47)	87.19 (85.85-88.53)	82.68 (81.01-84.35)
**Ethambutol**	85.97 (84.57-87.36)	80.74 (78.88-82.61)	85.78 (84.39-87.17)	80.56 (78.71-82.41)
**Ethionamide**	86.74 (85.41-88.08)	82.21 (80.49-83.94)	86.86 (85.57-88.15)	82.49 (80.85-84.13)
**Amikacin**	91.18 (90.21-92.15)	89.38 (88.26-90.49)	92.05 (91.23-92.86)	90.47 (89.54-91.41)
**Rifampicin**	94.6 (94.03-95.17)	94.31 (93.71-94.91)	94.8 (94.27-95.34)	94.57 (94.01-95.14)
**Isoniazid**	94.87 (94.37-95.38)	94.74 (94.2-95.29)	94.97 (94.47-95.48)	94.91 (94.37-95.45)
**Rifabutin**	95.6 (95.13-96.07)	92.75 (92.09-93.41)	95.65 (95.19-96.1)	92.86 (92.22-93.5)

S1 Table. Oligopeptide and oligonucleotide sample heritability estimates for binary resistant vs. sensitive phenotypes compared to semi-quantitiative MIC phenotypes. Sample heritability estimates and 95% confidence intervals are shown for the 13 drugs.
